# Managing complex trauma injuries in the elderly: a case report of a free flap and circular frame in a 95-year old patient with an open IIIB tibial fracture

**DOI:** 10.1007/s00238-018-1405-4

**Published:** 2018-03-20

**Authors:** Charlotte Hammonds, Philippa C. Jackson, Patrick Foster, Jonathan D. Wiper

**Affiliations:** 1 Leeds School of Medicine, University of Leeds, Leeds, England; 2 NHS Leeds Teaching Hospitals Trust, Leeds General Infirmary, Leeds, England

**Keywords:** Elderly, Trauma, Free flap, Circular frame

## Abstract

In an ageing population, increasing numbers of patients over the age of 70 are sustaining severe trauma. These patients require careful multidisciplinary team (MDT) management with careful consideration of existing co-morbidities, as such their treatment should be individually tailored. We present the case of a patient believed to be the oldest documented patient treated in a trauma setting with free flap and circular frame fixation to an open tibial fracture. A 95-year-old male presented to the Level 1 Major Trauma Centre (MTC) with multiple injuries after a pedestrian vs car incident. His injury severity score (ISS) was 22. For treatment of his open tibial fracture, he required soft tissue coverage with a free anterolateral thigh (ALT) flap, and circular frame application. Microsurgery was performed after consultation with the MDT and was uneventful. The circular frame was removed after 10 months and the patient went on to regain pre-injury mobility. Use of free tissue transfer in elderly patients is well documented in the elective setting, but less so in trauma. This case demonstrates that careful patient selection, attention to detail and MDT working can result in an excellent outcome for the patient. The challenges faced in treating this patient will be described in detail.

Level of Evidence: Level V, therapeutic study.

## Introduction

As the population of the UK ages, medical professionals will need to perform interventions on patients that we are yet to gain experience of. Free flap surgery is a common technique in the reconstruction of both traumatic and non-traumatic, soft tissue and bony defects. The use of microsurgery in the older population is well documented, particularly in oncological resection and reconstructions [1]. However, as the population ages, and with the rise in traumatic injuries in patients over the age of 70 [2], this will push microsurgery into new territory.

The bulk of the literature to date relates to post-oncological resection reconstruction in the elderly population. Two series recently published by Sierkowski et al. [3] and Mitchell et al. [4] concentrate on free flaps in the elderly (over 70 and 80 years, respectively). In these cases, the flap failure rate was 4.55 and 4.41%, respectively and the mortality rate was 0.96 and 1.51%, respectively. This highlights the complexity of managing these patients; the outcomes of flap failures are comparable to younger cohorts yet mortality is higher. This notion is supported by the findings of a meta-analysis conducted by Üstün et al. [5] where there were no significant differences in flap failure and surgical complications between the older and younger cohorts. However, Üstün et al. [5] do report a significant relationship between advanced age and mortality/systemic complications. Although there are obvious differences between elective oncological cases and traumatic lower limb cases, lessons can be learned: the use of frailty scoring systems prior to surgical intervention, limiting intraoperative times and minimising fluid shifts have all been suggested to minimise complications and mortality in the elderly [6].

The case that follows is of a 95-year-old male who required a free anterolateral thigh (ALT) flap for coverage of an open traumatic tibia and fibula fracture of the right leg. This gentleman also received a circular frame for treatment of his fracture. Review of the literature demonstrates that this is the oldest patient reported to receive such treatment in a trauma setting.

## Case report

**Fig. 1:**
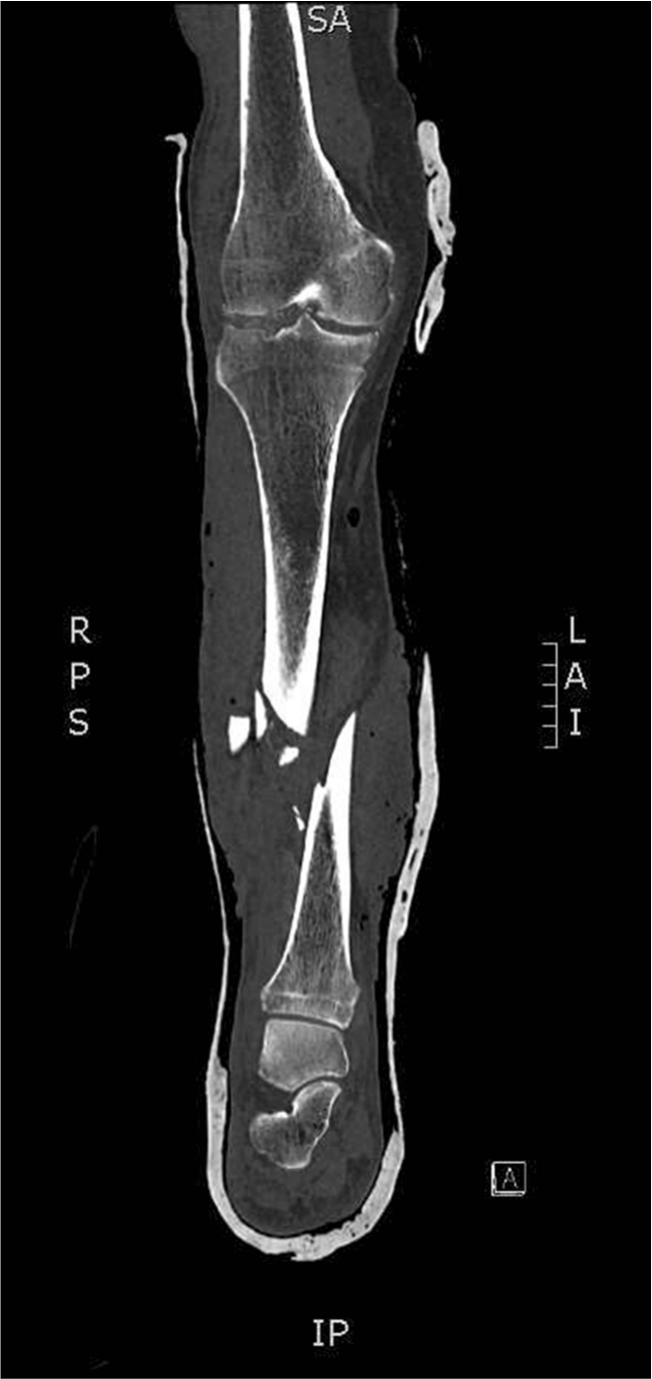
Trauma CT demonstrating comminuted mid-shaft tibial fracture with partial bone loss

A 95-year-old male presented to the Emergency Department of a Level 1 Major Trauma Centre (MTC) after being hit by a car and sustaining major trauma. The patient was previously fit and well with a past history of hypertension (American Society of Anaesthesiologists (ASA) Grade 2), and lived independently with an exercise tolerance of 700 yards without walking aids. Upon presentation, the patient underwent a complete trauma assessment and was found to have sustained the following injuries giving him an injury severity score (ISS) of 22—right flail chest with rib fractures 2–7, right scapular fracture, lumbar vertebral body fracture, sacral alar fracture, Rockwood grade 2/3 dislocation of the acromioclavicular joint (ACJ), Gustillo and Anderson grade IIIB open fracture with partial bone loss of the tibia and fibula (see Fig. 1), and an open right mid-foot fracture. The spinal, pelvic, scapular and rib fractures were managed non-operatively as was the ACJ dislocation.

Prior to undertaking any surgical intervention, the patient was treated in a high dependency (HDU) setting to manage his thoracic injuries. Surgery was required for the open tibial and foot fractures, and he underwent a total of four procedures for this. The first was application of an external fixator, debridement of the wound and temporisation with a negative pressure therapy dressing. The open foot fracture was debrided and primarily closed. As per the joint British Orthopaedic and Plastic Surgical guidelines, this procedure was performed with senior orthoplastic input within 24 h of the injury. Initial management was in line with BOAST4 protocol [7]. The second procedure on day 5 post-injury was primary tibial shortening following further bony debridement, and free ALT flap, following which the patient remained on HDU for 2 further days as per local protocol. On day 20, the patient had a circular frame applied to the leg with no adverse effects to the flap. Approximately 3 months after the frame was applied, the patient developed a pin site infection which required revision of the frame, which was the fourth operation. The pin site infection was felt to be due to loosening of the pins within osteopaenic bone and Exogen® treatment was commenced.

**Fig. 2:**
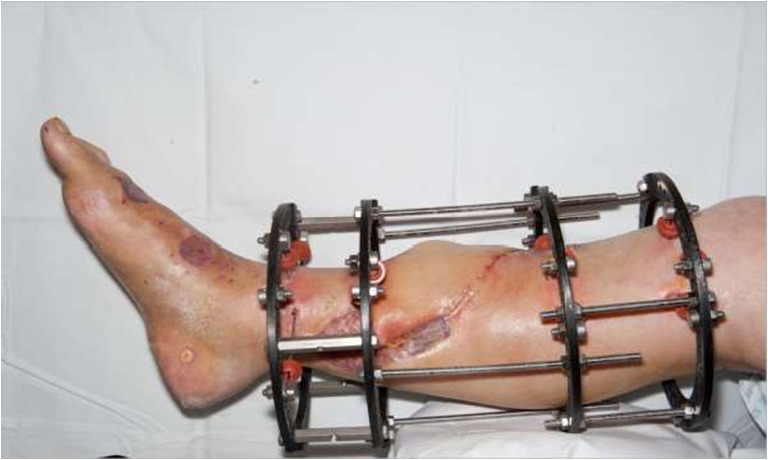
Two weeks following application of circular frame, maturing skin grafts and healthy free ALT flap

Soft tissue management in this case required free tissue transfer as local options were not available. Discussion within the multidisciplinary team, and with the patient, determined that the only alternative to free tissue transfer would be amputation. The left ALT flap was raised with a strip of vastus lateralis and the donor site was closed primarily. The flap was anastamosed end-to-side onto the posterior tibial artery, and end-to-end onto the venae commitantes (see Fig. 2). A small skin graft was required for the dorsum of the foot.

**Fig. 3:**
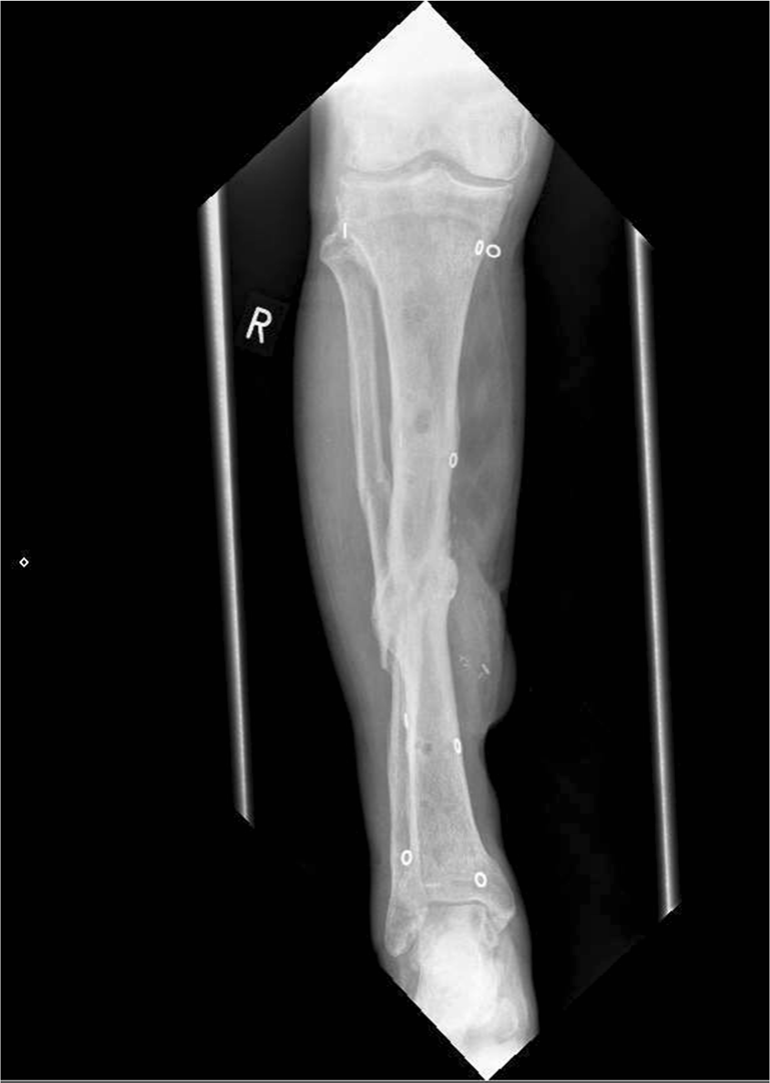
AP x-ray of the right lower leg following removal of circular frame demonstrating new bone formation and adequate alignment. Surgical clips at the site of flap anastomosis are visible on the medial surface

**Fig. 4:**
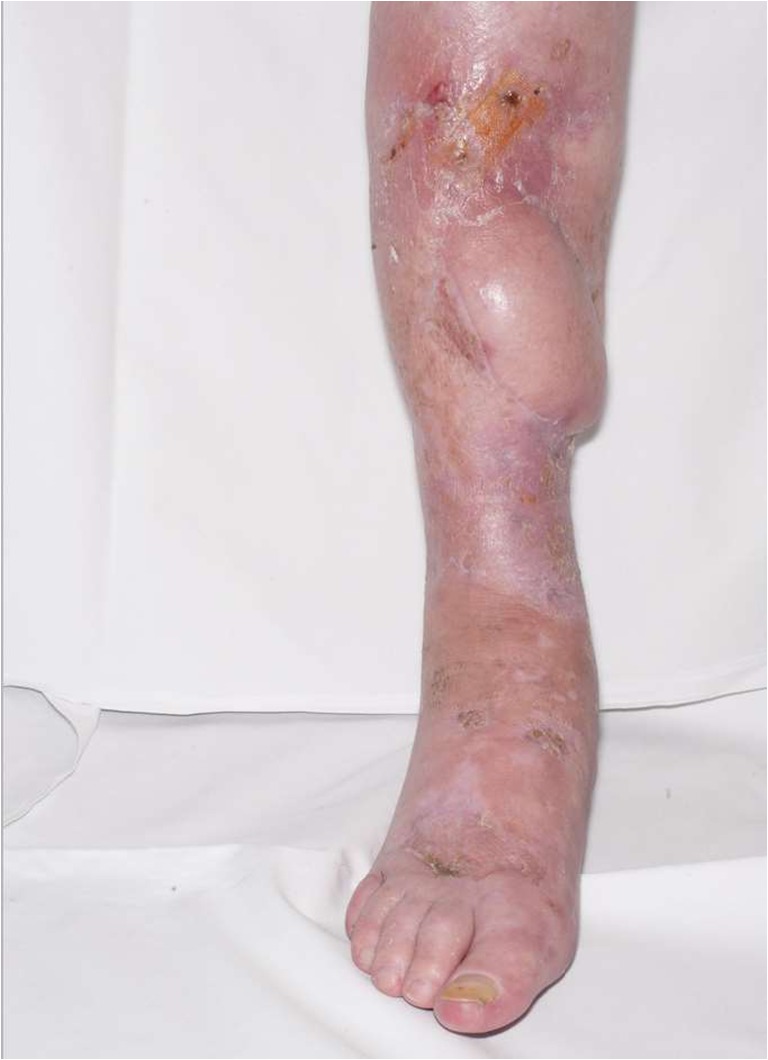
Two weeks following circular frame removal showing healing at the site of pin removal, and viable flap coverage and limb salvage

Following the acute event, the patient was discharged home once ambulatory and continued to live independently with assistance from his family. The frame was removed at 10 months (see Figs. 3 and 4) and at 15 months he weight-bears without a cast or boot and has resumed his pre-injury activities with excellent soft tissue cover.

## Discussion

We report the oldest patient in the literature to receive a free flap following trauma to the lower limb. At the age of 95, this gentleman sustained major trauma and his care during this episode required careful management with adjusted expectations of outcome and consideration of realistic goals.

A key part of this management was to ensure decisions were made with the patient and his family, and the extended multidisciplinary team. A holistic approach was taken to this patient’s care. Following initial stabilisation surgery, the patient was optimised prior to undertaking free flap surgery, which was slightly delayed in order to allow intensive chest physiotherapy. The decision to pursue limb salvage, as well as the method of fixation, was reviewed regularly during treatment.

Physiological demands on the patient to undergo free tissue transfer include not only peri-operative challenges, but more significantly post-operative challenges. In an elderly patient, the peri-operative challenges can be split into anaesthetic related, cardiovascular, and surgical. Pre-operative assessment by the anaesthetist performing the microsurgical procedure is crucial to permit request of relevant tests. Tools that were utilised by the team to assess the patient for fitness to undergo microsurgery included ASA grade, local Trauma questionnaire and WHO performance status but many other systems exist (ICED, ACE27, Charlson comorbidity index) [1, 3, 6, 8–10]. Cardiovascular challenges are predominantly experienced post-operatively when invasive monitoring of cardiac output is replaced by more crude measures such as urine output and pulse pressure. It is important to recognise that elderly patients are likely to function in a narrower physiological window when compared with the typical young male trauma patient. Therefore, this type of surgery may not be appropriate in less-robust patients, and in contrast to those elderly patients receiving free tissue transfer for oncological reconstruction in an elective setting, the impact of any trauma must not be underestimated in this group.

Surgical challenges in elderly patients with respect to soft tissue reconstruction will occur predominantly with technical aspects of the microsurgery. However, surgical preparation is crucial to minimise operative and anaesthetic time, and includes careful flap selection. The flap must be expendable, unlikely to cause further reduced mobility in the elderly, quick to raise and reliable. Choice of the recipient vessels may be aided with pre-operative imaging of the vasculature of the limb. This may indicate that pre-operative intervention such as angioplasty would be beneficial and this can be performed in an acute setting. It may also give an indication to the quality of the proposed recipient vessels. Ectatic vessels, or stripping of the adventitia, should be anticipated and dealt with by adjusting microsurgical technique and adapting plans where required. From the orthopaedic perspective, challenges were encountered with pin site infection as a result of loosening within poor quality osteopaenic bone. Although the circular frame permitted early weight-bearing mobilisation, and is generally well tolerated in the elderly [11], it required close follow-up and careful patient selection.

## Conclusions

This 95-year-old patient underwent successful limb salvage in the context of severe major trauma. Elderly patients should be assessed for suitability by an experienced multidisciplinary team to determine whether limb salvage is appropriate. Where microsurgery is being performed, all aspects of peri-operative care must be optimised to minimise operative and anaesthetic time. Technical adaptations may be required to deal with older anatomy and physiology. As more patients survive to older age, and the rate of trauma increases in the elderly, this is likely to be a more common occurrence and Major Trauma Centres must prepare for this group.
